# The loss of photosynthetic pathways in the plastid and nuclear genomes of the non-photosynthetic mycoheterotrophic eudicot *Monotropa hypopitys*

**DOI:** 10.1186/s12870-016-0929-7

**Published:** 2016-11-16

**Authors:** Nikolai V. Ravin, Eugeny V. Gruzdev, Alexey V. Beletsky, Alexander M. Mazur, Egor B. Prokhortchouk, Mikhail A. Filyushin, Elena Z. Kochieva, Vitaly V. Kadnikov, Andrey V. Mardanov, Konstantin G. Skryabin

**Affiliations:** Institute of Bioengineering, Research Center of Biotechnology of the Russian Academy of Sciences, Moscow, Russia

**Keywords:** Chloroplast genome, Parasitic plant, Mycoheterotrophy, Photosynthesis, Gene loss, Transcriptome

## Abstract

**Background:**

Chloroplasts of most plants are responsible for photosynthesis and contain a conserved set of about 110 genes that encode components of housekeeping gene expression machinery and photosynthesis-related functions. Heterotrophic plants obtaining nutrients from other organisms and their plastid genomes represent model systems in which to study the effects of relaxed selective pressure on photosynthetic function. The most evident is a reduction in the size and gene content of the plastome, which correlates with the loss of genes encoding photosynthetic machinery which become unnecessary. Transition to a non-photosynthetic lifestyle is expected also to relax the selective pressure on photosynthetic machinery in the nuclear genome, however, the corresponding changes are less known.

**Results:**

Here we report the complete sequence of the plastid genome of *Monotropa hypopitys,* an achlorophyllous obligately mycoheterotrophic plant belonging to the family *Ericaceae*. The plastome of *M. hypopitys* is greatly reduced in size (35,336 bp) and lacks the typical quadripartite structure with two single-copy regions and an inverted repeat. Only 45 genes remained presumably intact– those encoding ribosomal proteins, ribosomal and transfer RNA and housekeeping genes *infA*, *matK, accD* and *clpP*. The *clpP* and *accD* genes probably remain functional, although their sequences are highly diverged. The sets of genes for ribosomal protein and transfer RNA are incomplete relative to chloroplasts of a photosynthetic plant. Comparison of the plastid genomes of two subspecies-level isolates of *M. hypopitys* revealed major structural rearrangements associated with repeat-driven recombination and the presence of isolate-specific tRNA genes. Analysis of the *M. hypopitys* transcriptome by RNA-Seq showed the absence of expression of nuclear-encoded components of photosystem I and II reaction center proteins, components of cytochrome *b6f* complex, ATP synthase, ribulose bisphosphate carboxylase components, as well as chlorophyll from protoporphyrin IX biosynthesis pathway.

**Conclusions:**

With the complete loss of genes related to photosynthesis, NADH dehydrogenase, plastid-encoded RNA polymerase and ATP synthase, the *M. hypopitys* plastid genome is among the most functionally reduced ones characteristic of obligate non-photosynthetic parasitic species. Analysis of the *M. hypopitys* transcriptome revealed coordinated evolution of the nuclear and plastome genomes and the loss of photosynthesis-related functions in both genomes.

**Electronic supplementary material:**

The online version of this article (doi:10.1186/s12870-016-0929-7) contains supplementary material, which is available to authorized users.

## Background

Heterotrophic plants obtaining nutrients from other organisms, either directly from other plants (direct parasites) or through mycorrhizal fungi (mycoheterotrophs) with which they associate, often display a reduction in morphological and genomic features. Some heterotrophic plants retain photosynthetic capabilities being hemiparasites, while others completely lose the ability to carry out photosynthesis (i.e. holoparasites). Plastid genomes of parasitic plants represent model systems in which to study the effects of relaxed selective pressure on photosynthetic function. The most evident is a reduction in the size and gene content of the plastid genome, which correlates with the loss of genes encoding photosynthetic machinery which becomes unnecessary [1–3]. Nearly all heterotrophic plants demonstrate a reduction of their plastomes compared with photosynthetic relatives, ranging from minimal in some “early” parasites such as *Corallorhiza striata* [4] and *Cuscuta* sp. [5], to extreme in some endoparasitic species like *Pilostyles aethiopica* [6], and possibly even the complete loss of the plastome in *Rafflesia lagascae* [7].

Transition to a non-photosynthetic lifestyle is expected to relax the selective pressure on photosynthetic machinery encoded not only in the chloroplast, but also in the nuclear genome. However, the corresponding changes in the nuclear genome and transcriptome of parasitic plants are less known and limited to few studies (e.g. [8, 9]). For example, transcriptome analysis of a holoparasitic plant *Phelipanche aegyptiaca* of the family *Orobanchaceae* revealed an expected loss of expression of photosynthesis-related genes and surprising conservation and transcription of the chlorophyll biosynthesis pathway [10].

Mycoheterotrophy evolved independently in at least 50 plant lineages comprising approximately 400 species [11]. Most of the full mycoheterotrophs are monocots, primarily representing the *Orchidaceae* family [12, 13]. Relatively few complete plastid genomes of mycoheterotrophic plants have been sequenced, including the orchid species *Corallorhiza striata, Epigogium roseum*, *Epigogium aphyllum, Neottia nidus-avis* and *Rhizanthella gardneri* [4, 14–16], *Petrosavia stellaris* [17], *Sciaphila densiflora* [18], and the liverwort *Aneura mirabilis* [19]. Among eudicots, mycoheterotrophs were found in the family *Ericaceae* [11], which belongs to the order *Ericales* of Asterids [20]. Many species of *Ericaeae* forms associations with mycorrhizal fungi and obtain nutrients from them. It is supposed that mycoheterotrophic plants have evolved from photosynthetic mycorrhizal lineages under low-light conditions , where they loss photosynthetic capabilities and established tight association with fungi, which penetrate the roots of green trees and provided the sufficient amounts of carbon and nutrients to its mycoheterotrophic partner [12, 21].

*Monotropa hypopitys* (pinesap) is an achlorophyllous obligately mycoheterotrophic plant of the family *Ericaceae*, subfamily *Monotropoideae* (reviewed in [22]). The above-ground part of *M. hypopitys* plant is 5–35 cm tall unbranched adventitious raceme-like inflorescence with 2 to 11 flowers on the top and scale-like bracts which cover most of the inflorescence (Additional file 1: Figure S1). Short roots are invested by a net of sheathing mycorrhizas forming haustorium-like structures used to attach the fungal partner. Recently we sequenced the plastid genome of *M. hypopitys* isolate MON2-KALR [23], the first reported plastome of mycogheterotrophic eudicots. The plastid genome is only 34800-bp long; it is highly rearranged relative to the gene order typical of most the other plastomes of angiosperms, lacking the quadripartite structure with two inverted repeats regions. The reduction in size correlates with the loss of genes encoding NADH dehydrogenase, photosynthesis-related proteins, the plastid-encoded RNA polymerase and some other functions [23]. The loss of photosynthesis-related genes in mycoheterotrophic *Ericaceae* was also previously shown by Braukmann and Stefanovic [24] using a slot-blot Southern hybridization approach.

Our previous studies revealed the substantial genetic diversity within *M. hypopitys* and the possible existence of two distinct subspecies or varieties [25, 26]. Taking into account the accelerated evolution of the chloroplast genomes in parasitic plants, existence of such close but distinct subspecies provides an opportunity for a comparative analysis of the molecular changes that mark the progression of plastid genome degradation. Here we report the full plastome sequence of the second subspecies isolate of *M. hypopitys*, MON-1VOLR and present a comparative analysis of plastomes of both subspecies. In addition, in this study we analysed the transcriptome of *M. hypopitys* to assess the changes in the nuclear genome associated with a transition to mycoheterotrophy and the loss of the ability to perform photosynthesis.

## Methods

### Plastid genome sequencing and annotation

A single *Monotropa hypopitys* plant was collected from the Vologda region, Russia (58°35’14” N, 37°59’20” E). The specimen is stored under accession number MON-1VOLR. Total genomic DNA was extracted from fresh leaves of a single individual and sequenced with a Roche Genome Sequencer GS FLX (Roche, Switzerland) using the Titanium XL+ protocol for a shotgun genome library. About 86 Mb of sequences with an average read length of 356 bp were generated. De novo assembly was performed with Newbler Assembler v. 2.9 (454 Life Sciences, Branford, USA), which yielded three chloroplast DNA contigs with 18-fold coverage. These contigs were identified based on sequence similarity to chloroplast genomes of related species (*Ericales*) and high coverage. The complete plastid genome sequence was obtained upon the generation of appropriate PCR fragments covering the gaps between the contigs and their sequencing by Sanger method on ABI PRISM 3730 (Applied Biosystems). To verify the correct assembly of the reconstructed plastid genome, raw reads were mapped against the reconstructed sequence with GS Reference mapper (454 Life Sciences, Branford, USA). Plastid genome annotation was performed using DOGMA [27] with further manual correction. Circular map of the plastome was drawn using OrganellarGenomeDRAW tools [28].

The sequence of the plastid genome of M. *hypopitys* MON-1VOLR was submitted to GenBank under accession number KU640957.

### Transcriptome sequencing and assembly

Leaves (flower bracts) and flowers of two individual *Monotropa hypopitys* plants (not the same specimen used for chloroplast genome sequencing) and two pooled samples of roots with haustoria were collected for transcriptome analysis. Total RNA was extracted from ~0.3 g tissue for each six samples using the RNeasy Plant Mini kit (Qiagen, Valencia, CA). RNA samples were sequenced using the Illumina HiSeq2500 platform (100-bp reads) according to the manufacturer’s instructions (Illumina Inc., USA). RNA-seq read data has been deposited in the NCBI SRA database under accession SRP069226.

*M. hypopitys* transcriptome sequencing resulted in a total of 103 million high quality sequencing reads after primer and quality trimming with Cutadapt [29] and Sickle (https://github.com/najoshi/sickle), respectively. Assembly of the transcriptome from the combined six RNA-seq datasets was carried out using the Trinity platform [30]. The transcriptome was assembled into 98350 unigenes ranging from 201 to 12993 bp in length with N50 of 1342 bp. Coding region identification in Trinity assembly was done using TransDecoder (http://transdecoder.github.io). Trinotate (https://trinotate.github.io/) was used to assign hits from TrEMBL and Swiss-Prot databases (http://www.uniprot.org/uniprot/), and to assign GO terms and pfam domains (accessed 15/02/2016). 37977 unigenes were annotated in the TrEMBL protein database using predicted protein sequences and 38419 unigenes were annotated in the Swiss-Prot database using BLASTX). Protein-coding genes were assigned with the KEGG orthology identifiers using web-based KAAS server [31].

The levels of transcription of protein-coding genes in the plastid genome were quantitated employing RSEM [32] and Bowtie 2 program [33]. Transcription levels were expressed as fragments per kilobase of exon per million fragments mapped (FPKM) values.

## Results and discussion

### Plastome size, gene content and expression

**Fig. 1:**
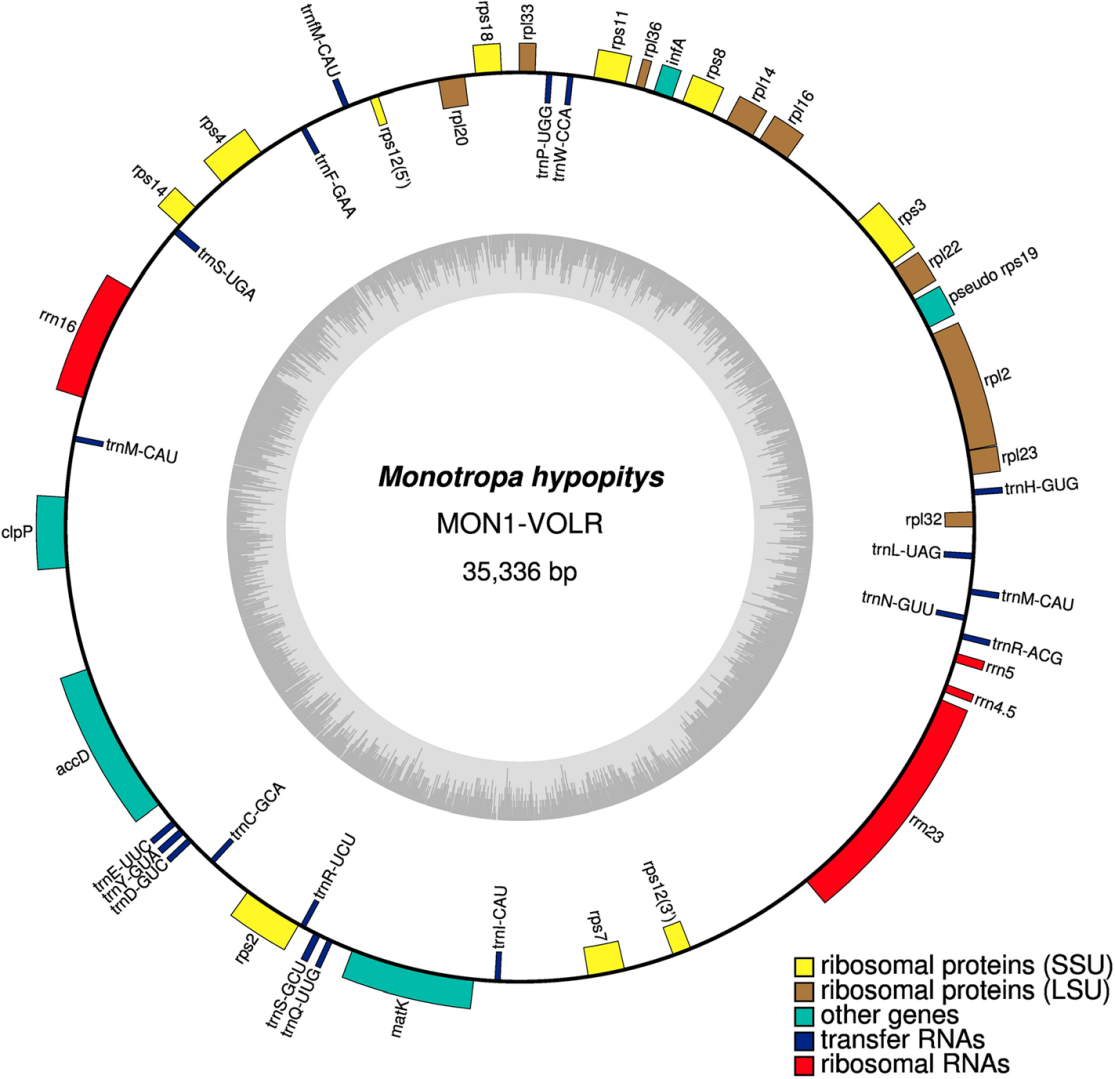
Circular map of the plastid genome of *M. hypopitys* MON1-VOLR. Genes shown inside the circle are transcribed clockwise, those outside the circle are transcribed counterclockwise. Asterisks indicate intron-containing genes, dark gray bars inside the inner circle indicate guanine-cytosine content

The plastid chromosome of *Monotropa hypopitys* MON1-VOLR is only 35,336 bp in length and lacks a typical quadripartite structure with two single-copy regions and an inverted repeat (Fig. 1). This is one of the most highly reduced plastid genome known from nonphotosynthetic angiosperms after those of *Pilostyles* sp (11–15 kb, [6]), *Sciaphila densiflora* (21 kb, [18]), and *Epigogium* sp. (19–31 kb, [16]). The *M. hypopitys* MON1 plastome is predicted to contain 45 presumably intact genes (Table 1). Most of these genes are involved in protein synthesis: four ribosomal RNA genes, 19 transfer RNA genes, nine genes code for small subunit and nine for large subunit ribosomal proteins. The remaining genes are *infA* (translation initiation factor), *matK* (the maturase for splicing of group IIA introns), *accD* and *clpP*.

**Table 1:** Summary of genes retained in the *M. hypopitys* plastome

Function	Genes
Ribosomal proteins (large subunit)	*rpl2, rpl14, rpl16, rpl20, rpl22, rpl23, rpl32, rpl33, rpl36*
Ribosomal proteins (small subunit)	*rps2, rps3, rps4, rps7, rps8, rps11, rps12, rps14, rps18*
rRNAs	*rrn4.5, rrn5, rrn16, rrn23*
tRNAs	*trnH-GUG, trnW-CCA, trnP-UGG, trnfM-CAU* ^*a*^ *, trnF-GAA, trnS-UGA, trnM-CAU* ^*b*^ *, trnE-UUC, trnY-GUA, trnD-GUC, trnC-GCA, trnR-UCU, trnS-GCU, trnQ-UUG, trnI-CAU, trnR-ACG, trnN-GUU, trnL-UAG, trnF-AAA* ^*c*^
Other protein-coding genes	*infA, matK, clpP, accD* ^*d*^

^a^ two copies in MON1 and one copy in MON2 plastome

^b^ missing in MON2 plastome

^c^ missing in MON1 plastome

^d^ functionality is unclear

**Fig. 2:**
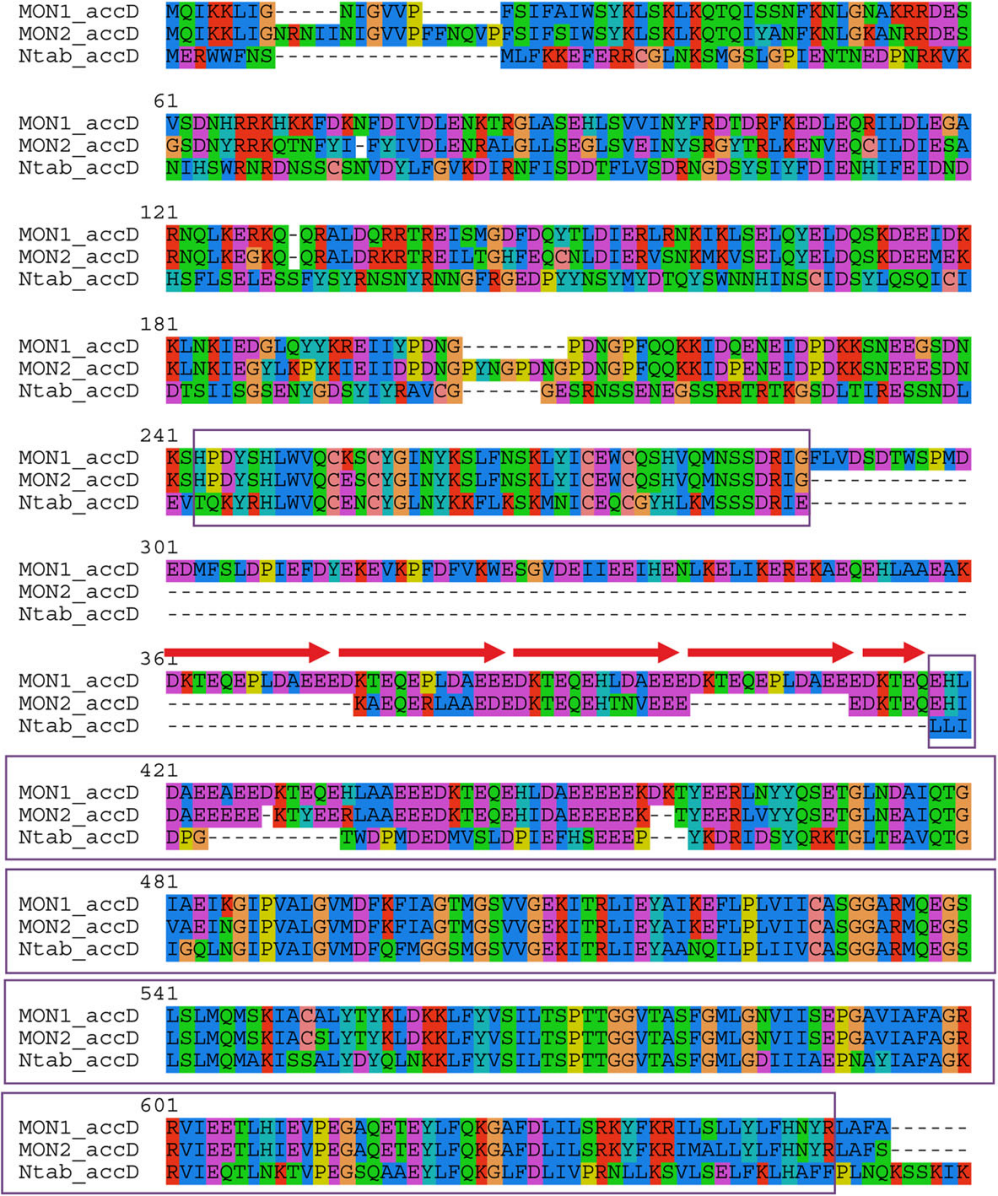
Alignment of predicted amino acid sequences of AccD proteins from *M. hypopitys* MON1-VOLR (MON1-accD), *M. hypopitys* MON2-KALR (MON2-accD) and *Nicotiana tabacum* (Ntav_accD). Repeats in the MON1-accD sequence are shown by arrows. The AccD conserved domain (COG0777) is boxed. Note that it is interrupted by in-frame insertions in both *M. hypopitys* plastomes

The functionality of the *accD* gene coding for the β-subunit of the acetyl-CoA carboxylase is unclear since its deduced protein product is very diverged in the N-terminal region and has long repeat-containing internal insertion relative to typical AccD proteins (e.g. from *Nicotiana tabacum*, Fig. 2). However, the insertions do not interrupt the open reading frame. AccD is involved in fatty-acid synthesis and leaf development [34] and is supposed to be essential to plastome maintenance [35]. Most plastomes of parasitic plants contain a functional copy of the *accD* gene; however, in *Phelipanche* it is also highly diverged, containing long indels and appears to lack a standard start codon [36].

Like *accD*, the *clpP* gene is supposed to be essential for plastid maintenance and is present even in strongly reduced plastomes of parasitic plants [14]. *clpP* encodes a proteolytic subunit of Clp protease involved in the import of proteins into the plastid [35]. *M. hypopitys* MON1 plastome the *clpP* gene lacks both introns and has a very diverged protein sequence with an N-terminal extension, although the whole functional domain (pfam00574) is present (Additional file 2: Figure S2). The closest relative of this protein is ClpP from the chloroplast of the eudicot *Asclepias nivea* with only 33 % amino acid sequence identity. It should be noted that accelerated evolution of ClpP and intron losses was observed in several parasitic and photosynthetic angiosperm lineages (for example, [36, 37]).

The *rps19* is likely a pseudogene judging from its 5’-terminal truncation and the lack of start codon associated with an accumulation of short tandem repeats in this region (Additional file 3: Figure S3). The functionality of *rpl22* is unclear since the predicted protein product of this gene is shorter than its homologs in related species due to C-terminal truncation (130 a.a. vs 154–159 a.a.), although the full Rpl22 conservative domain (pfam00237/CHL00034) is retained. Genes for ribosomal proteins Rps15 and Rps16 are missing like in many other plastomes of parasitic plants [28, 36].

Consistently with the obligate mycoheterotrophy of *M. hypopitys*, its plastome lacks all genes coding for the thylakoid NADH-dehydrogenase complex, photosynthesis-related proteins (photosystems I and II, cytochrome *b*_*6*_ *f* complex, rubisco large subunit and ATP synthase). The genes for plastid-encoded RNA polymerase, photosynthesis-related gene *ycf1* [38] and conserved genes *ycf2, ycf3* and *ycf4* are also missing.

Reduction of the size and gene content of the *M. hypopitys* plastome is also reflected in the loss of introns. Only a single intron was found in the *rpl2* gene, while introns in *rpl16* and *clpP* are missing. The *rps12* is a trans-splicing gene as in most other angiosperms, but it consists of only two rather than three exons indicating the lack of one intron. The presence of *matK* maturase in spite of strong reduction of the plastome genome could be determined by the retention of the group IIA intron in the *rpl2* gene which requires maturase activity for processing [39].

We analyzed expression of predicted protein-coding genes in the *M. hypopitys* plastome using RNA-seq data from above-ground part of a single plant. The reads were mapped to the plastid genome and the number of reads corresponding to protein-coding genes (exons) were calculated and normalized per kilobase of the gene length. A total of 33,659 reads were mapped to the protein coding genes. Expression was observed for all genes (Table 2) and, interestingly, the most highly expressed genes were *accD* and *clpP* with FPKM value >100,000. In contrast, *matK* and *rpl32* showed lower expression (FPKM value <10,000).

**Table 2:** Transcription of protein coding genes in the *M. hypopitys* MON1-VOLR plastid genome

Gene	Position (start-end)	FPKM value
*rpl32*	195-1	4424
*rpl23*	650-949	36165
*rpl2*	954-1349, 2032-2460^a^	66036
*rpl22*	3007-3399	71151
*rps3*	3465-4136	92022
*rpl16*	5308-5718	98751
*rpl14*	5817-6185	86138
*rps8*	6383-6781	75817
*infA*	6916-7155	83289
*rpl36*	7278-7391	105328
*rps11*	7519-7920	27030
*rpl33*	8642-8845	29301
*rps18*	9060-9386	36275
*rpl20*	9855-9517	29237
*rps12*	28675-28433, 10730-10617^b^	97942
*rps4*	12232-12843	56329
*rps14*	13291-13593	89745
*clpP*	17294-18169	102855
*accD*	19448-21352	261840
*rps2*	22894-23646	37404
*matK*	24380-25930	4868
*rps7*	27814-27347	155291

^a^ two exons

^b^ two exons, trans-splicing

### Comparison of two plastid genomes of *M. hypopitys* revealed extensive rearrangements

**Fig. 3:**
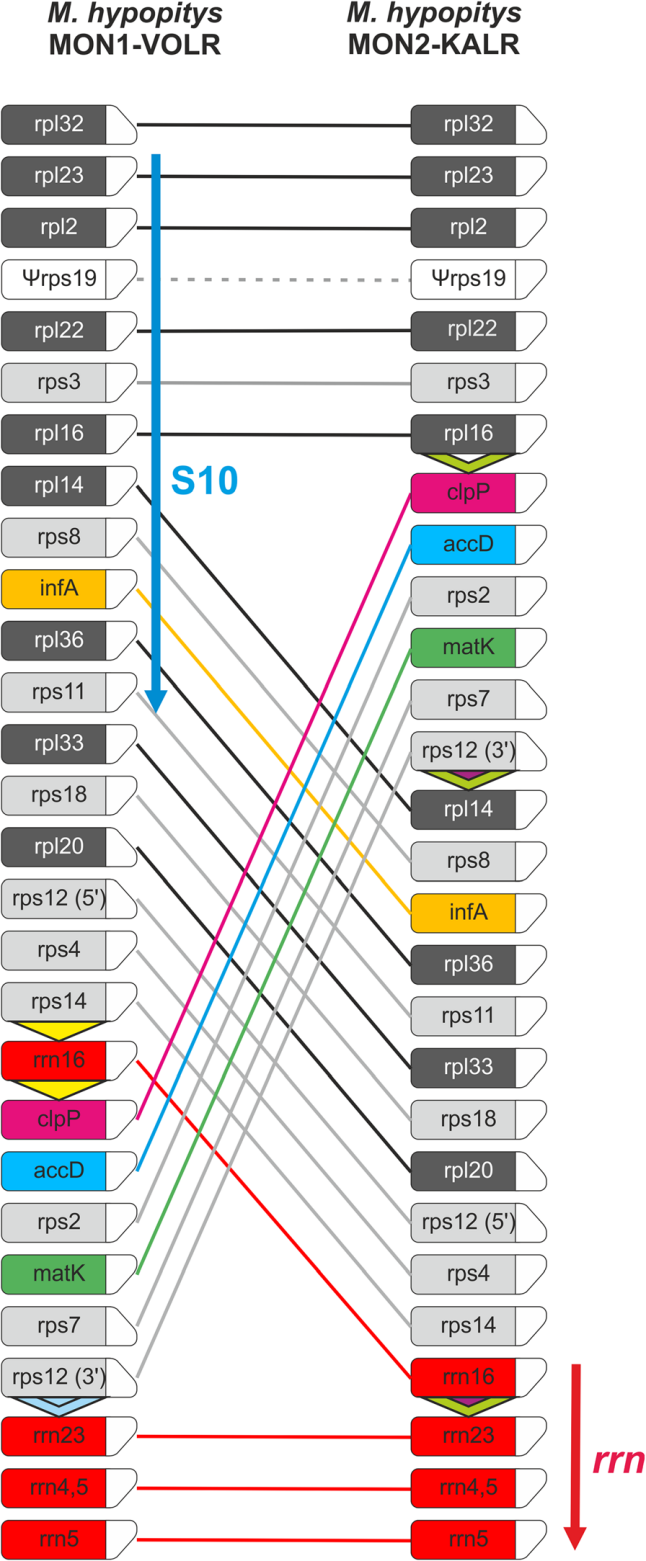
Schematic gene order comparison maps between the plastid genomes of *M. hypopitys* MON1-VOLR and *M. hypopitys* MON2-KALR. The linear representation of the circular mapping genomes using *rpl32* genes as the starting point does not reflect the actual gene size or spacing between the coding regions. Boxes represent protein-encoding and rRNA genes with the direction of transcription shown by the chamfered top corner. Triangles in *M. hypopitys* maps indicate repeat sequences. The S10 and *rrn* operons are shown by arrows. Note that comparison of the structures of plastid genomes of *M. hypopitys* and *Nicotiana tabacum* is shown in Additional file 4: Figure S4

The availability of the second complete plastome genome of *M. hypopitys*, obtained from another subspecies-range sample, termed MON2-KALR [23], allowed us to study evolutionary events at a short time scale by comparative analysis. Surprisingly for a single species, these two plastid genomes not only differ slightly in length (35,336 bp for MON1 vs 34,800 bp for MON2 genome), but also show differences in gene order and content (Fig. 3).

The genomes differ in a single major rearrangement and several local minor indels. The major difference is an exchange of positions of genome regions comprising, according to the *M. hypopitys* MON1 plastome map, genes *rps14-rrn16* and *clpP- rps12* (5’-exon). In both genomes gene order significantly deviates from the order conserved in photosynthetic angiosperms (Additional file 4: Figure S4) and none could be considered as an “ancestral” form. The first genome, MON1, retains an intact structure of the highly conserved S10 operon (*rps11, rpl36, infA, rps8, rpl14, rpl16, rps3, rpl22, rps19, rpl2, rpl23*) which is broken in the second genome by the rearrangement. In contrast, the *M. hypopitys* MON2 plastome contains *rrn16-rrn23-rrn4.5-rrn5* gene cluster, which is split in the MON1 genome (Fig. 3). The structures of the S10 and ribosomal RNA operons are highly conserved in the chloroplast genome since it ensured co-expression of the components required for assembly of the ribosome. Despite numerous rearrangements in the course of transition to the non-photosynthetic lifestyle, the gene order in the S10 and *rrn* operons is conserved even in such highly reduced genomes as in *Sciaphila* and *Epigogium* sp. [16, 18].

The rearranged regions are flanked by a repeat sequence that could facilitate recombination events [40, 41]. Three such repeats were found in the MON2 plastome at the “junction” points marking the rearranged regions (Fig. 3); an exchange of the fragment located between the first and the second repeat with one between the second and the third in the MON2 plastome would produce the observed MON1 gene plastome structure.

The content of protein-coding genes is the same in both *M. hypopitys* plastomes, although some genes whose functionality is questionable are quite variable. The predicted protein products of *rpl22* differ in length and both are shorter than in related *Ericales* species. The deduced products of *accD* both have N-terminal extensions and internal insertions of different size and low similarity (Fig. 2). The predicted *clpP* gene in the MON2 plastome contain tandem 72-bp long imperfect duplications in its 5’ region (Additional file 2: Figure S2), but the common parts of the encoded proteins are highly similar (87 % identity) suggesting conservation of functionality. While this article was in review, a report describing the plastid genomes of *Monotropa uniflora* and another isolate of *M. hypopitys* was published [42]. It confirmed our previous findings regarding the reduction of the *M. hypopitys* plastid genome [23], and provided evidences that *clpP* and *accD* are intact in these species.

Surprisingly, the two plastid genomes encode a similar but not identical inventory of tRNAs. The MON1 plastome encodes 19 tRNA species for 15 amino acids, and the second plastome encodes 16 tRNA for 14 amino acids. Two tRNA genes are present only in MON1 and one only in the MON2 plastome (Table 1). Interestingly, the second plastome encodes trnF-AAA which is unusual for plant chloroplast genomes and has been reported previously in the parasitic plant *E. roseum* [16].

Recently Barrett and Davis proposed a model of chloroplast genome degradation in course of transition to heterotrophy [4]. According to this model plastid genes are lost in the following order: NADH dehydrogenase, genes responsible for photosynthesis (*psa, psb, pet, rbcL*), the plastid-encoded RNA polymerase and ATP synthase genes. Genes encoding ribosomal RNAs and ribosomal proteins, transfer RNAs and several other essential genes (*accD, clpP, matK, ycf1, ycf2*) are the last to be lost. The gene content of the *M. hypopitys* plastid genome indicates that this plastome is close to the end of the reduction process since it lacks even some housekeeping genes (*ycf1, ycf2,* some tRNAs and ribosomal proteins). The notable exceptions are *clpP* and *accD* genes which probably remains functional, although their sequences are highly diverged. High levels of transcription of these genes in the plastome also support their functionality. The AccD and ClpP are the components of multi-subunit protein complexes that must be assembled within the chloroplast and thus the *clpP* and *accD* genes are retained in the plastomes [14]. The presence of genes coding for other subunits of Clp protease in the *M. hypopitys* transcriptome (to be published elsewhere) further supports this hypothesis.

### The loss of nuclear photosynthesis-related genes, as revealed by transcriptome analysis

The structure and gene content of the *M. hypopitys* plastid genome clearly shows the complete loss of genes related to photosynthesis. Therefore, we analysed whether the loss of photosynthesis function is accompanied by the loss of the corresponding genes encoded at the nuclear genome. To answer this question we analysed the presence of the photosynthesis-related genes in the *M. hypopitys* transcriptome using the *Arabidopsis* reference pathways as a query [10]. Among 63 genes of the KEGG pathway “Photosynthesis” only three were detected in the transcriptome (Additional file 5: Table S1). Of the photosystem I and II reaction center proteins, components of cytochrome *b*_*6*_ *f* complex, ATP synthase and ribulose bisphosphate carboxylase components, none was detected in the transcriptome, indicating gene losses and/or absence of expression. Sequencing and analysis of the nuclear genome of *M. hypopitys* will answer the question whether these genes were lost or only silenced.

Unlike the components of the photosynthetic reaction centers, expression of the chlorophyll biosynthesis genes was previously detected in non-photosynthetic parasitic plant *P. aegyptiaca* [10]. However, analysis of the *M. hypopitys* transcriptome revealed that among the chlorophyll biosynthesis pathway components [43] only a single gene, divinyl chlorophyllide a 8-vinyl-reductase, was expressed at a low level and only in flowers, while protoporphyrin IX Mg-chelatase, protoporphyrin IX methyltransferase, Mg-protoporphyrin IX monomethyl ester oxidative cyclase, protochlorophyllide oxidoreductase and chlorophyll synthase were absent from the transcriptome. These findings are consistent with the lack of chlorophyll in *M. hypopitys* [44]. The upstream biosynthetic pathway generating protoporphyrin IX from aminolevulinic acid is present and expressed, as expected since these steps are common for both heme and chlorophyll synthesis.

## Conclusions

With the complete loss of genes related to photosynthesis, namely NAD(P)H dehydrogenase, plastid-encoded RNA polymerase and ATP synthase, the *M. hypopitys* plastid genome is among the most functionally reduced ones among obligate non-photosynthetic parasitic species. Gene loss and genome reduction were associated with rearrangements, loss of the typical quadripartite structure of the plastid genome and also the loss of introns. The advanced process of plastid genome degradation also involved some housekeeping functions as evidenced by the loss of some ribosomal protein and transfer RNA genes. Comparison of the plastid genomes of two subspecies-level isolates of *M. hypopitys* revealed major structural rearrangements associated with repeat-driven recombination and the presence of isolate-specific tRNA genes. Analysis of the *M. hypopitys* transcriptome revealed the coordinated evolution of the nuclear and plastid genomes and the loss of photosynthesis-related functions in both genomes.

## Additional files

Additional file 1:
**Figure S1.** *M. hypopitys* plants in its natural environment. (PDF 436 kb)
Additional file 2:
**Figure S2.** ClpP coding regions. (PDF 97 kb)
Additional file 3:
**Figure S3.** Nucleotide sequences of putative rps19 pseudogene and upstream intergenic regions. (PDF 68 kb)
Additional file 4:
**Figure S4.** Schematic gene order comparison maps between the plastid genomes of *M. hypopitys* MON1-VOLR, *M. hypopitys* MON2-KALR and *Nicotiana tabacum*. (PDF 238 kb)
Additional file 5:
**Table S1.** Analysis of the presence of genes of the KEGG pathway “Photosynthesis” in the transcriptome. (XLS 29 kb)
