# Supplementary figures and images for: The loss of photosynthetic pathways in the plastid and nuclear genomes of the non-photosynthetic mycoheterotrophic eudicot Monotropa hypopitys
Source: BMC Plant Biol. 2016 Nov 16;16(Suppl 3):153–61. doi: 10.1186/s12870-016-0929-7 (PMC5123295; doi:10.1186/s12870-016-0929-7)

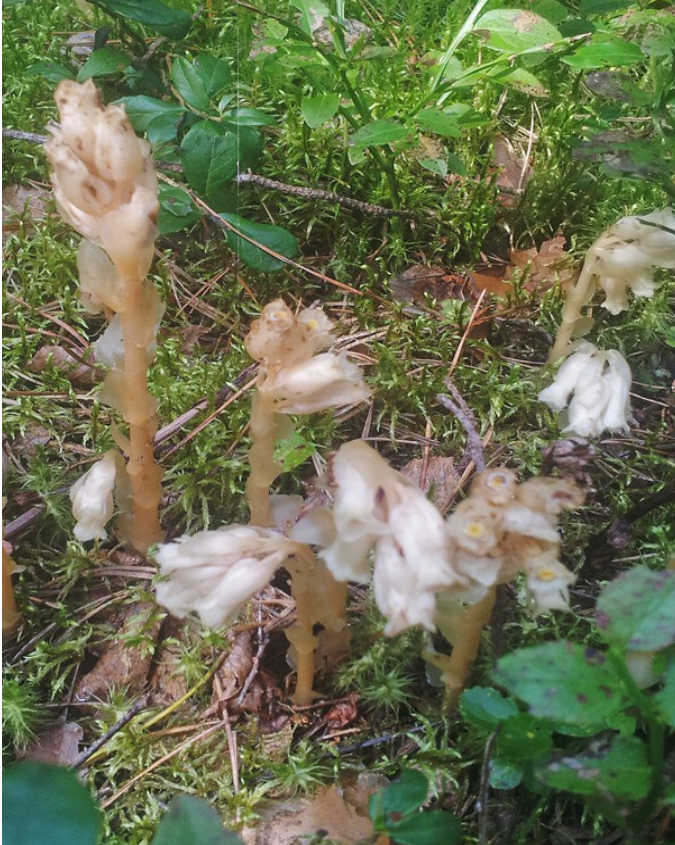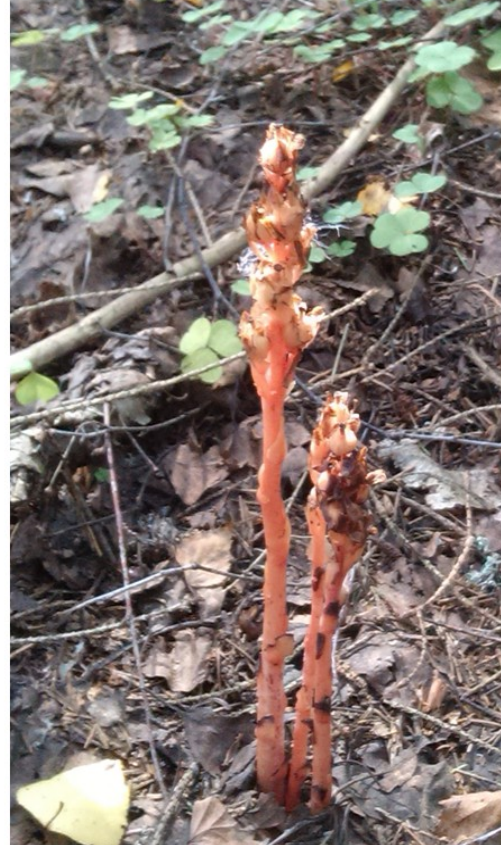

Figure S1. *M. hypopitys* plants in its natural environment.

Supplement: Additional file 1: — Figure S1. M. hypopitys plants in its natural environment. (PDF 436 kb) [file 12870_2016_929_MOESM1_ESM.pdf]
