# Supplementary material for: The loss of photosynthetic pathways in the plastid and nuclear genomes of the non-photosynthetic mycoheterotrophic eudicot Monotropa hypopitys
Source: BMC Plant Biol. 2016 Nov 16;16(Suppl 3):153–61. doi: 10.1186/s12870-016-0929-7 (PMC5123295; doi:10.1186/s12870-016-0929-7)
Supplement: Additional file 2: — Figure S2. ClpP coding regions. (PDF 97 kb) [file 12870_2016_929_MOESM2_ESM.pdf]

(A)

```
  1  MIFYRNYINA DPFDDPFDDE GDEKDKKGKR KKKEKKEPKE PKIIIPKVPY IEIEEVEELE
 61  RIENEPNDSE NGDEDEDEAE IIYEKKIYYE EVVNFIDLYS FLYKNGIIFL WENLTMEVAN
121  KIIKLIICLD IYGDATETAS VLINCSKGSL LASRTLCNFM NEGSDINIET IGCGQVGGPG
181  IYALLGGRTR LAFPNCSFLL SRPNIKLDPR LPPSEYEVDA NIKLQICGSL QDIFTEKTGQ
241  PPEFIRDIGR RVRYMSAAEA REYGIIDEII RGFNFKKPED ETQEKSDKNG E
```

(B)

```
1
MON1_ClpP ATGATTTTTTATCGAAATTACATAAACGCAGATCCTTTTGACGATCCTTTTGACGATGAA
Mon2_ClpP ATGATTTTTGATCGAAATTACATAAACGCGGATCCTTTTGACGATCCTTCTGACGATGAA
61
MON1_ClpP GGGGATGAAAAGGATAAAAAGGGTAAAAGGAAGAAGAAGGAGAAAAAAGAGCCTAAAGAG
Mon2_ClpP GGGGATCTCGAAAAGGGAAGAAAAAGGAAGAAAAGGAGAAAACCTAAAGAGCCTAAAGAG
121
MON1_ClpP CCTAAAATAATTATCCCTAAAGTTCCTTATATAGAAATAGAAGAAGTTGAAGAGTTAGAA
Mon2_ClpP CCTAAAATAATTATCCCTAAAGTTCCTTATATAGAAATAGAAGAAGTTGAAGAGTTCGAA
181
MON1_ClpP AGAATTGAAAATGAACCTAATGACAGTGAAAATGGGATGAGGA-----------------
Mon2_ClpP AGAATTGAAAATGAACCTAATGACAGTGAGTATGAGTATGAGGAAGAAATAGACGAAGTT
241
MON1_ClpP ------------------------------------------------------------
Mon2_ClpP GAAGAGTTCGAAAGAATTGAAAATGAACCTAATGACAGTGAGTATGAGTATGAGGAAGAA
301
MON1_ClpP ------------------------------------------------------------
Mon2_ClpP ATAGACGAAGTTGAAGAGTTCGAAAGAATTGAAAATGAACCTAATGACAGTGAGTATGAG
361
MON1_ClpP ------------------------------------------------------------
Mon2_ClpP TATGAGGAAGAAATAGACGAAGTTGAAGAGTTCGAAAGAATTGAAAATGAACCTAATGAC
421
MON1_ClpP --TGAGGATGAGGCAGAAATAATTTATGAAAAAAAAATATATTATGAAGAAGTAGTAAAT
Mon2_ClpP AGTGAGGCTGAGGAAGAAATAATTTAT-AAAAAAATATATATTATGAAGAAGTAGTAAAT
481
MON1_ClpP TTTATTGATTTATATTCTTTTCTTTATAAGAACGGTATTATATTTCTATGGGAAAATCTC
Mon2_ClpP TATATTGATTTAGATTCTTTTCTTTATAAGAAAGGTCTTATCTTTCTATGGGAAAATCTC
541
MON1_ClpP ACTATGGAGGTCGCTAATAAAATTATAAAATTGATAATATGCCTTGATATTTATGGTGAT
Mon2_ClpP ACTATGGAGGTCGCTAATAAAATTATACAATTGATAATATGCCTTGATATTTATGGTGAT
601
MON1_ClpP GCGACGGAAACGGCAAGCGTTCTTATAAACTGCTCTAAAGGAAGCCTACTTGCTTCAAGG
Mon2_ClpP GCGCCAGAAAAGGCAAGTGTTCTTATAAACTGCTCTAAGGGAAGCCTACTTGCTTCAAGG
661
MON1_ClpP ACTCTTTGTAATTTCATGAATGAGGGGTCAGACATAAACATAGAGACAATAGGCTGCGGG
Mon2_ClpP ACTCTTTGTAATTTCATGACTGAGGGGGCAGACATAAACATAGAGACAATAGGATGTGGA
721
MON1_ClpP CAGGTTGGAGGACCGGGAATCTATGCCTTGCTTGGGGGAAGGACACGTTTAGCATTCCCT
Mon2_ClpP CAGGTTGGAGGACCGGGAATCTATACCTTGCTTGGGGGAAGGACACGTTTTGCATTCCAT
781
MON1_ClpP AACTGTTCGTTCCTGCTTTCTCGACCGAACATTAAATTGGATCCGCGTCTTCCACCCTCA
Mon2_ClpP AACTGTAGGTTCCTGCTTTCCCGACCGAACATTAAATTTGATCCACGTCTTCCGCCCTCA
841
MON1_ClpP GAATATGAAGTTGATGCAAACATAAAATTGCAGATATGTGGATCTCTACAAGATATTTTT
Mon2_ClpP GAATATGAGGTTGATGCAAACATAAAATTGGAGATATGTAGGGGTCTAGAAGATATTTTT
901
MON1_ClpP ACGGAAAAAACGGGGCAACCTCCTGAATTTATAAGAGATATAGGAAGAAGAGTGCGTTAT
Mon2_ClpP ACGGAAAAAACGGGGCAACCTCCTGAATTTATAAGAGATATAGGAAGAGG---TTATTCT
961
MON1_ClpP ATGTCCGCGGCAGAAGCCCGAGAGTACGGAATTATCGACGAGATCATACGTGGTTTTAAT
Mon2_ClpP ATATCCGCGACAGAAGCCCGAGAGTACGGAATTATCGACTCGATCATACGTGGTTTTAAT
1021
MON1_ClpP TTTAAAAAACCTGAGGATGAAACCCAGGAAAAATCAGACAAAAATGGGGAATAA
Mon2_ClpP TTTAAAAAACCTGAGGATGAAACCCAGGAAAAAGAAGACAAAAATGGAGAATAA
```

Figure S2. ClpP coding regions.

(A) Predicted amino acid sequence of the ClpP protein from *M. hypopitys* MON1-VOLR. The ClpP protease domain (pfam00574) is shaded.

(B) Alignment of the nucleotide sequences of ClpP coding regions from *M. hypopitys* MON1-VOLR (MON1-ClpP) and *M. hypopitys* MON2-KALR (MON2-ClpP) plastomes. 72-nt long sequence, tandemly duplicated in the MON2 (4 copies), is shaded and shown by an arrow. Note that there is a frameshift in the *clpP* reading frame in MON2 genome due to 1 bp deletion at bp 448, but it was not verified.
