# Supplementary material for: The loss of photosynthetic pathways in the plastid and nuclear genomes of the non-photosynthetic mycoheterotrophic eudicot Monotropa hypopitys
Source: BMC Plant Biol. 2016 Nov 16;16(Suppl 3):153–61. doi: 10.1186/s12870-016-0929-7 (PMC5123295; doi:10.1186/s12870-016-0929-7)
Supplement: Additional file 3: — Figure S3. Nucleotide sequences of putative rps19 pseudogene and upstream intergenic regions. (PDF 68 kb) [file 12870_2016_929_MOESM3_ESM.pdf]

M. hypopitys MON1 plastome

```
2461    atatagtgataatttgattcttcgtcgtcgtaaatagaaaataaattatatttctctatt
2521    catttatcttaaaaaaaatgggggggaggaagctgtgacagactcactaaaggaaagcc
2581    ttttgtaagaaagccctatgtaagaaagccctatgtaagaaagcctttgtaagaaagcc
2641    ctatgtaagaaagccctatgtaagaaagccctatgtactaaaaaaaagggattttgtagc
2701    tcttcatttattaaaaaaaattgaaaagcttaaaacaaaaaaagaaataataagaacttg
2761    gtcccgggcatctaccattatacccgaaatggtcggccatactattgctattcataatgg
2821    aaaagagcatttacctatcttcataaaagcagttatgctaggtcataaattgggagaatt
2881    ttcgcctacgtgtaatttcctagaccatgggaaaggtgataagcgatctcgtcgttaa
```

M. hypopitys MON2 plastome

```
2538                               gaagagaaattagatttatatatttatcttaaaaaaagggagga
2581    agctatgacagactcacttaaaagaagtccctatgtaagaaagccctatgtaagaaagcc
2641    ctatgtactaaaaaaaagggattttgtagctattcatttattaaaaaaattgaaaggctta
2701    aaacaaaaggaaagggaaataataataacttggtcccgggcatctactattatacccgaa
2761    atggttggccatactattgctattcataatggaaaagagcatttacctatctttataaaa
2821    gcagttatgataggtcataaattgggagaatttgcacctacgcgaaatttcctagaacat
2881    ggtaaaggtgagaagcgatctcgtcgttaa
```

Figure S3. Nucleotide sequences of putative rps19 pseudogene and upstream intergenic regions. Coordinates are shown according to the sequences of *M. hypopitys* MON1-VOLR and *M. hypopitys* MON2-KALR plastomes. GTAAGAAAGCCnTnT repeat is shown by arrows. Grey shading indicates regions exhibiting sequence similarity to Rps19 of *Arbutus unedo* (AEX37346) revealed by BLASTX search (+ strand is shown). Note that these regions contain intact open reading frames but there are no recognizable start codons upstream.
