# Supplementary material for: The loss of photosynthetic pathways in the plastid and nuclear genomes of the non-photosynthetic mycoheterotrophic eudicot Monotropa hypopitys
Source: BMC Plant Biol. 2016 Nov 16;16(Suppl 3):153–61. doi: 10.1186/s12870-016-0929-7 (PMC5123295; doi:10.1186/s12870-016-0929-7)
Supplement: Additional file 4: — Figure S4. Schematic gene order comparison maps between the plastid genomes of M. hypopitys MON1-VOLR, M. hypopitys MON2-KALR and Nicotiana tabacum. (PDF 238 kb) [file 12870_2016_929_MOESM4_ESM.pdf]

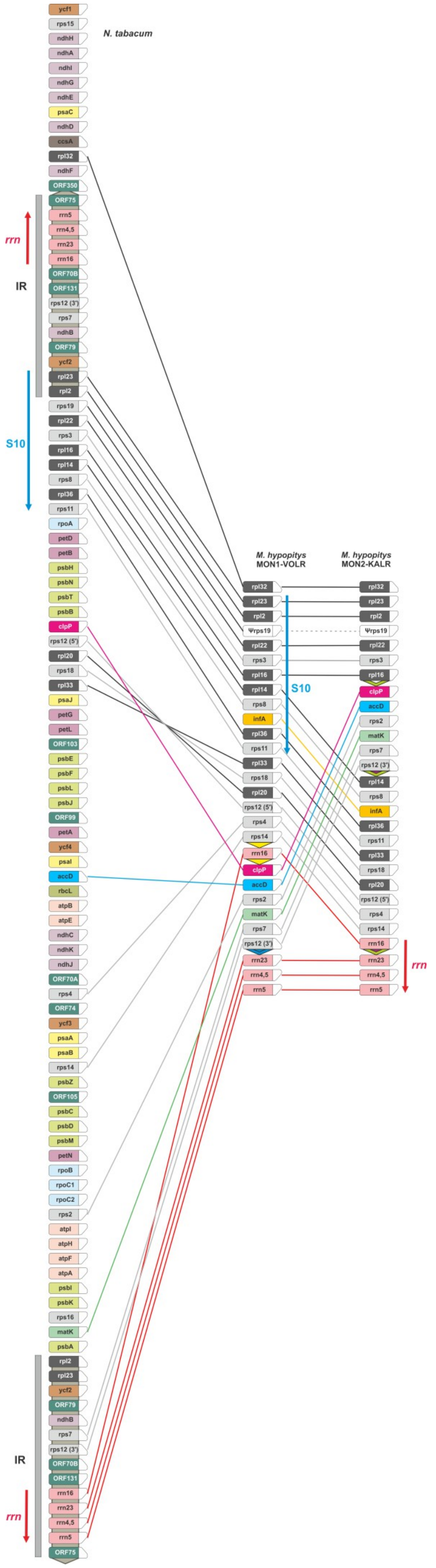

Figure S4. Schematic gene order comparison maps between the plastid genomes of *M. hypopitys* MON1-VOLR, *M. hypopitys* MON2-KALR and *Nicotiana tabacum*.

The linear representation of the circular mapping genomes using *rpl32* genes as the starting point does not reflect the actual gene size or spacing between the coding regions. Boxes represent protein-encoding and rRNA genes with the direction of transcription shown by the chamfered top corner. Triangles in *M. hypopitys* maps indicate repeat sequences. The S10 and *rrn* operons are shown by arrows. Locations of IR regions in *N. tabacum* chloroplast genome are shown by gray rectangles.
